# The Real Culprit in Systemic Lupus Erythematosus: Abnormal Epigenetic Regulation

**DOI:** 10.3390/ijms160511013

**Published:** 2015-05-15

**Authors:** Haijing Wu, Ming Zhao, Christopher Chang, Qianjin Lu

**Affiliations:** 1 Department of Dermatology, Second Xiangya Hospital, Central South University, Hunan Key Laboratory of Medical Epigenomics, Changsha 410011, China; E-Mails: chriswu1010@126.com (H.W.); zhaoming307@126.com (M.Z.); 2 Division of Rheumatology, Allergy and Clinical Immunology, University of California at Davis, Davis, CA 95616, USA; E-Mail: c3chang@yahoo.com

**Keywords:** SLE, epigenetics, DNA methylation, histone modification, microRNA

## Abstract

Systemic lupus erythematosus (SLE) is an autoimmune disease involving multiple organs and the presence of anti-nuclear antibodies. The pathogenesis of SLE has been intensively studied but remains far from clear. B and T lymphocyte abnormalities, dysregulation of apoptosis, defects in the clearance of apoptotic materials, and various genetic and epigenetic factors are attributed to the development of SLE. The latest research findings point to the association between abnormal epigenetic regulation and SLE, which has attracted considerable interest worldwide. It is the purpose of this review to present and discuss the relationship between aberrant epigenetic regulation and SLE, including DNA methylation, histone modifications and microRNAs in patients with SLE, the possible mechanisms of immune dysfunction caused by epigenetic changes, and to better understand the roles of aberrant epigenetic regulation in the initiation and development of SLE and to provide an insight into the related therapeutic options in SLE.

## 1. Introduction

Systemic lupus erythematosus (SLE) is a multi-systemic, autoimmune disorder that predominately affects women (the female to male ratio is 9 to 1) during their reproductive years [1]. The prevalence and incidence of SLE have been shown to vary across geographic regions around the world. It has been found more frequently in non-white populations compared to Caucasians, and the highest prevalence is reported among Afro-Caribbeans [2]. Asian populations also have higher incidence and prevalence compared to Caucasians [3].

SLE is characterized by a presence of a diverse set of autoantibodies in the blood of affected patients [4], together with auto-reactive T and B lymphocytes [5,6]. Although the direct cause of SLE remains unidentified, many factors are believed to contribute to autoimmunity in SLE, including genetic susceptibility, epigenetic, hormones and environmental factors [7]. SLE occurs when an individual with genetic susceptibility to lupus encounters environmental triggers such as sunlight, drugs or infection. Immune tolerance is broken down such that T cells recognize self-antigens and provide help to auto-reactive B cells, which produce abundant autoantibodies. These autoantibodies bind to self-antigens and reside in multiple organs, leading to organ inflammation, dysfunction and failure. Various organ systems can be affected by SLE, including skin, joint, kidney, central nervous system and bone marrow [8,9,10,11].

## 2. Epigenetics and Systemic Lupus Erythematosus (SLE)

Epigenetics, the study of the reversible and potentially heritable changes in gene expression without genetic code alterations, including DNA methylation, histone modification and microRNA (miRNA), is a new area of investigation into the pathogenesis of SLE [12]. Indeed, epigenetics provides an additional explanation for how genetics can contribute to health and disease, and is being increasingly recognized as an important factor in the pathogenesis of SLE. For example, only 20% of concordance for SLE has been found in homozygotic twins, suggesting that there are roles of environmental and epigenetic factors in the onset of SLE [13]. Further indirect evidence of the role of epigenetics is that SLE is found predominantly in females and that it can be accelerated by environmental triggers (e.g., infection, UV, drugs) or/and internal factors (hormones and stress). In addition, certain types of drugs, which have been reported to induce SLE, are also known to cause epigenetic modifications, including 5-azacytidine and procainamide [14]. However, the precise mechanism by which these and many other environmental agents initiate lupus flares in genetically predisposed individuals has not been elucidated. This review summarizes up-to-date data showing the immune abnormalities induced by epigenetic changes in SLE patients and provides insight into the possibility of epigenetic therapies.

### 2.1. DNA Methylation and SLE

**Figure 1:**
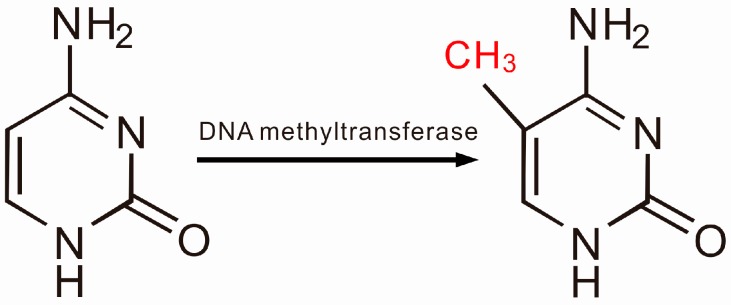
The process of DNA methylation.

**Figure 2:**
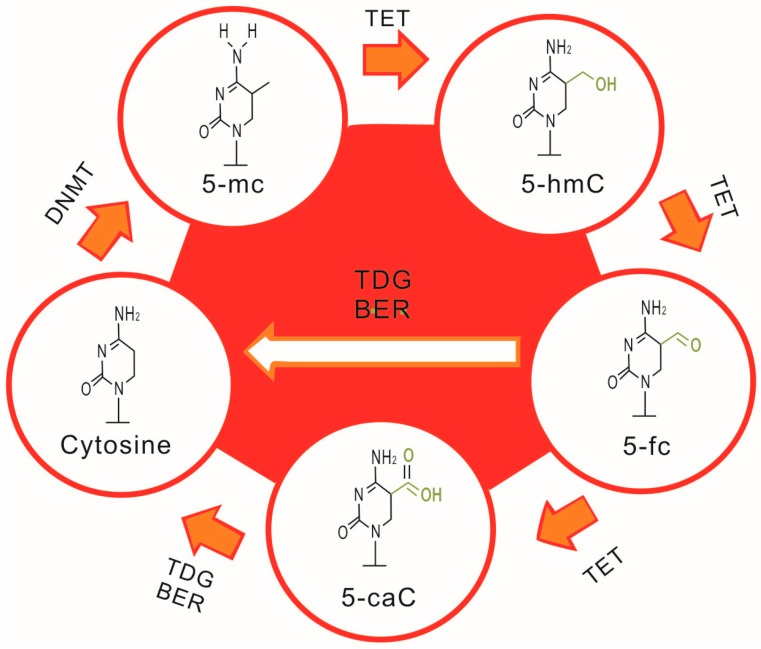
The cycle of DNA methylation and demethylation.

DNA methylation is a biochemical process in which a methyl group is added to a cytosine or adenine at the 5' position of a CpG dinucleotide, converting the cytosine to methyl-cytosine [15] (Figure 1). When present, this methylation serves as a mark that indicates repression of gene expression, and DNA methylation is therefore involved in many biological processes, such as cell differentiations and immune responses. The process of DNA methylation is regulated by methyltransferases, such as DNMT1, DNMT3a and DNMT3b, and each displays capacity different functional capacity. For example, DNMT1 maintains the methylation status during cell replication, whereas DNMT3a and 3b usually induce *de novo* methylation [16]. In contrast, DNA demethylation re-activates or re-expresses silenced genes, a process that is also regulated by enzymes, such as TET1, TET2 and TET3 (Figure 2). In mammalian cells, DNA methylation is restricted to regions of high CpG dinucleotide content, also known as CpG islands, which are typically present in promoter regions [17].

The role of DNA methylation in SLE first attracted attention in the 1960s and has since become a very hot research topic [18]. Two epigenetic drugs, procainamide and hydralazine, which are inhibitors of DNA methylation, were found to induce a lupus-like disease after long-term administration in wild-type mice, and the disease disappeared after the withdrawal of these drugs. To extrapolate this information to humans, DNA demethylation has been found in SLE CD4^+^ T cells, but not in CD8^+^ T cells, and peripheral blood mononuclear cells (PBMCs) [19,20]. However, the global DNA methylation status may not reflect actual specific gene expression patterns, but may instead indicate the activation status of cells in general. Thus, the most recent research has focused on specific cell types as well as certain SLE relevant genes.

### 2.2. Aberrant DNA Methylation, T Cell Abnormalities and SLE

T helper (Th) cells, as a major component of the adaptive immune system, have a critical function of “helping” B cells in the production of antibodies. Th cells are not a single population but can be divided into several subsets, such as Th1, Th2, Th17, Th9, Th22, follicular T (Tfh) and regulatory T (Treg) cells, based on their unique cytokines and transcription factors [21]. More importantly, these different subsets of Th cells are not automatically differentiated; their developmental trajectories from naïve T cells depend on the antigens presenting on major histocompatibility complex (MHC) molecules on dendritic cells or/and macrophages as well as the cytokine milieu provided by antigen-presenting cells (APCs). These processes occur in T cell zones in secondary lymphoid tissues after naïve T cells leave the thymus [22]. A mild dysregulation of any type of Th cells can result in severe immune disorders. It has been well documented that circulating CD4^+^ T cells are flexible; an imbalance of Th17 and Treg, Th1 and Th2, or even increased levels of Tfh, cells may contribute to SLE [23,24,25,26]. In addition to the unique transcription factors of these cells, recent studies have revealed that DNA methylation plays a role in controlling this sophisticated differentiation process.

Previously, Richardson *et al.* reported that DNA hypomethylation on lupus CD4^+^ T cells is associated with T cell auto-reactivity in lupus [27]. That study was the first attempt to explore the role of epigenetics in lupus, and it has prompted the coming of a new era of epigenetics in the study of pathogenesis. Their finding was supported by the induction of auto-reactivity of healthy CD4^+^ T cells by the addition of 5-aza-cytidine [27,28], which followed prior evidence of the induction of IL-2 and IFN-γ by the same agent [29]. An increasing number of studies have focused on the role of DNA methylation in the activation and differentiation of T cells at specific loci in individual genes. IFN-γ and IL-4 are well-described cytokines for Th1 and Th2 processes, respectively, and impaired DNA methylation has been found at *Ifng* and *IL4* loci during Th1 and Th2 differentiation [30,31]. DNA hypomethylation is observed at the *FOXP3* locus in regulatory T cells compared to naïve T cells [32]. In addition to these linage-specific cytokines and transcription factors, other genes such as HTR1A [33], LFA-1 [34], CD70 [35,36], perforin [37], and CD40L [38] have been found to be demethylated in lupus CD4^+^ T cells and have been associated with the pathogenesis of SLE. These genes, which are also related to auto-reactivity of T cells, are over-expressed in conjunction with DNA hypomethylation.

CD40L on T cells is a ligand for CD40, which is expressed on B cells and DCs. The joining of the CD40L-CD40 pathway provides the “secondary signal” to B cells or/and DCs, promoting the activation of these cells, which then may lead to a higher production of antibodies [39]. Recently, upstream factors of DNA methylation have also been explored. Zhao *et al*. observed that RFX1, a regulation factor for the X-Box protein family, is decreased in SLE and regulates DNA methylation in CD4^+^ T cells [35]. In addition, the level of DNA damage-inducible 45α (Gadd45α), which is thought to have demethylation capability, is reportedly enhanced in lupus patients and positively correlates with CD11a/CD70 expression [40]. Furthermore, high mobility group box protein 1 (HMGB1) has been found to be involved in DNA methylation by binding to Gadd45a [41]. In a large-scale DNA methylation investigation, DNA hypomethylation was found in interferon-regulated genes in lupus naïve T cells, including IFI44L, IFIT1, IFIT3, MX1, STAT1, USP18, BST2 and TRIM22, suggesting abnormalities in T cell progenitors [42]. Lupus-associated and inflammatory cytokine genes, such as *IL4*, *IL6*, *IL10* and *IL13*, are demethylated in lupus T cells and may contribute to disease progression [32,43,44]. Although it is known that epigenetics plays a critical role in T cell differentiation in general, our knowledge of how this impacts abnormalities in T cell differentiation in specific autoimmune diseases such as SLE is incomplete. However, further studies on the epigenetic regulation in specific pathogenic mechanisms known to occur in SLE are required before specific epigenetic treatments can be applied to the clinical setting.

In addition to T cell differentiation, epigenetic abnormalities may play a role in the various signaling pathways that are involved in SLE. Richardson *et al*. found that blocking the ERK signaling pathway in lupus CD4^+^ T cells resulted in the down-regulation of DNMT1 activity and DNA demethylation in daughter cells [37]. The auto-reactivity of ERK inhibitor (hydralazine)-treated CD4^+^ T cells was confirmed by the finding that injection into syngeneic mice caused lupus-like symptoms, similar to the 5-aza-cytidine treatments [45]. In addition, the inhibition of PKCδ, which is a step in the ERK pathway (PKC-ras-raf-MEK-ERK), causes hypomethylation of the CD70 promoter and enhances CD70 expression, resembling that seen in lupus or 5-aza-cytidine treated T cells [46,47]. More convincing evidence of ERKs role in the pathogenesis of SLE is shown in PKCδ “knock-out” mice which develop lupus-like symptoms [48,49], as well as dnMEK mouse model with autoimmune phenotype by inhibiting ERK signaling [50], indicating a critical role of this signaling pathway and the subsequent potential effect of epigenetic changes.

### 2.3. Environment and DNA Demethylation of T Cells in SLE

It is generally believed that DNA methylation or demethylation does not occur spontaneously, *i.e.*, there must be a trigger or external factor that leads to changes in DNA methylation. Many environmental factors have previously been implicated as triggers of SLE, and it is becoming more apparent that the mechanism by which these environmental insults exert their effects is through epigenetics. For example, oxidative stress, which may be caused by UV light, smoking, infections, mercury exposure and even air pollution, is capable of lowering DNMT1 levels in T cells [51]. Other external factors which can act synergistically include dietary deficiencies in Vitamin B, folate, methionine (Met), choline and Zn [52], all of which have been shown to be necessary to maintain a normal level of DNMT1 [53]. More and more environmental agents are being targeted as potential triggering factors for autoimmunity, but only a subset of these truly have a causal relationship. Further studies are necessary to delineate which environmental factors are true triggers of autoimmune disease and how epigenetics may play a role in pathogenesis.

### 2.4. Aberrant DNA Methylation, B Cell Abnormalities and SLE

As the source of autoantibodies, B cells have a direct and central role in the pathogenesis of SLE. Therefore, a growing number of clinical trials have targeted B cell therapies, without a complete understanding of the mechanisms leading to SLE. DNA methylation is clearly involved in B cell differentiation, as it is in T cells as described in the above section. For example, at the early stage of B cell differentiation, PAX-5, and Pu-1 regulation regions are demethylated [54,55]; in contrast, CD19 promoter demethylation occurs in pre-pro-B cells, and the CD21 promoter is demethylated in mature B cells [56]. However, in contrast to T cell, fewer studies have addressed DNA methylation in B cell abnormalities in SLE.

DNA demethylation has been observed in lupus B cells, especially in CD5-nonexpressing (B2) cells [57], promoting the auto-reactivity of B cells. Renaudineau *et al.* also detected an alteration in the regulation of HRES1/p28 expression in lupus B cells by DNA methylation [58]. In addition, direct evidence of DNA hypomethylation in B cells at the onset on SLE was provided by treating B cells *ex vivo* with DNMT1 inhibitors and then adoptively transferring them into syngeneic mice, which developed then anti-nuclear antibodies [59]. Although much evidence showing the DNA hypomethylation in V (D) J and lgh 3'-LCR, which contribute to autoantibody production [60,61], little has been revealed in this aspect in SLE. As the epigenome revolution unfolds, additional evidence will be available in the near future to reveal the factors that regulate DNA methylation in lupus B cells and how these demethylated genes contribute to SLE.

It has also been postulated that DNA methylation in X chromosomal silencing may serve as a potential explanation for the sex bias in lupus [62]. Moreover, dendritic cells (DCs) have also been implicated in the pathogenesis of SLE [63,64,65]. It is believed that the role of DCs in a normal functioning immune system involves their ability to clear apoptotic debris, recognize self DNA and RNA via Toll-like receptor (TLR) 9 and 7 expression in their cytoplasm, promote B cell maturation and proliferation through IFN-α secretion and polarize naïve T cells into different effector T cells via the cytokine milieu and surface markers they provide. Aberrations in the behavior of B cells through DNA methylation may play a role in the activation of B cells against self-tissues. Unfortunately, to this date, little research has been focused on the DNA methylation of DCs in lupus. Indeed, investigations have so far been restricted to hypomethylation- and methyltransferase-level observations for certain genes and the global levels of DNA methylation in lupus T and B cells (summarized in Table 1). With the advent of the epigenetic era, additional informative results, especially in a linage-specific manner, will be available in the near future.

**Table 1:** DNA methylation status in immune cells of lupus.

Cell Subsets, Molecules, or Genes	Change	Reference
Global methylation status in T and B cells	Decreased	[27,38,66]
DNMT expression in CD4^+^ T cells: DNMT1, DNMT3a, DNMT3b	Decreased	[38,66,67,68]
GADD45a in T cells	Increased	[40]
Methylation status of co-stimulatory molecules: CD6, CD11a, CD70, CD40L, CD5	Decreased	[34,57,69,70,71]
Methylation status of cytokine genes: *IL4*, *IL6*, *IL10*, *IL13*	Decreased	[32,43,44]
Methylation status of pro-inflammatory genes: *IFGNR2*, *MMP14*, *LNC2*, *CSF3R*, *AIM2*, *IFI44L*, *IFIT1*, *IFIT3*, *MX1*, *STAT1*, *USP18*, *BST2*, *TRIM22*	Decreased	[42]
Methylation status of HERV element LINE-1 in CD4^+^ T, CD8^+^ T and B cells	Decreased	[72,73]

## 3. Histone Modifications and SLE

**Figure 3:**
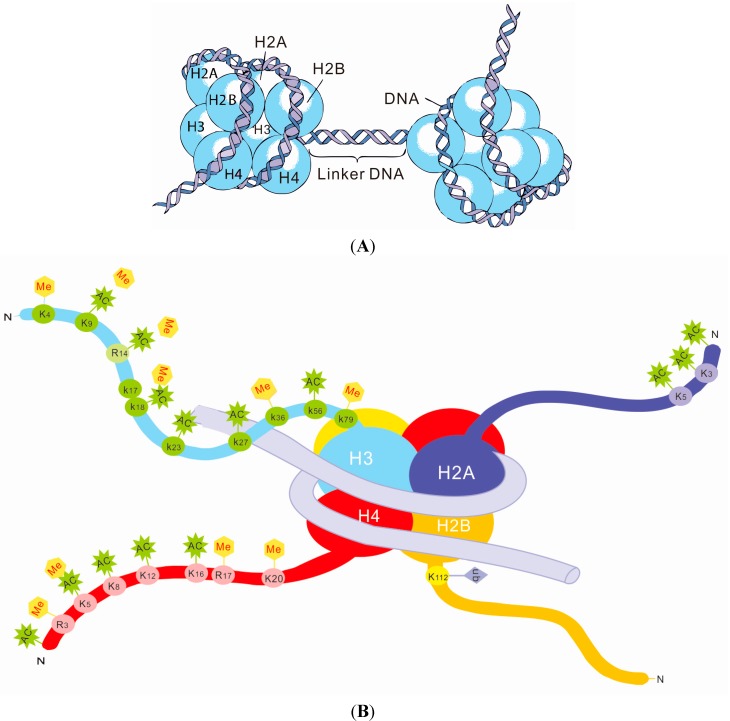
The structure and modifications of histones: (**A**) the structure of histones; and (**B**) the various modifications of histones. Me: Methylation; AC: Acetylation; Ub: Ubiquitination.

Histone modification is another important epigenetic mechanism for regulating gene expression. DNA is packaged into the nucleus as chromatin, with the nucleosome being the basic subunit of chromatin. Each nucleosome is formed by 146 base pairs (bp) of two turns of DNA wrapped around a histone core and contains two copies each of H2A, H2B, H3 and H4 (Figure 3A). The histones present small protein tails protruding from the nucleosome, which is accessible to modifications, including methylation, acetylation, and ubiquitination [74] (Figure 3B). Each modification has a specific function. For example, histone H3K9 acetylation enhances transcription, whereas methylation suppresses it. Among these modifications, acetylation and deacetylation has been intensively studied and is catalyzed by histone acetyltransferase (HAT) and histone deacetylase (HDAC), respectively. HAT transfers acetyl groups to lysine and leads to gene activation; HDAC removes acetyl groups, causing gene silencing [75]. Unlike acetylation, histone methylation occurs on arginine or lysine residues and is regulated by histone methyltransferase (HMT) and other enzymes, and the effects of methylation are subject to both the position of the modified residue and the number of methyl groups. It is well known that the addition of H3K4me3 enhances gene expression, whereas H3K9me3 and H3K27me3 lead to gene downregulation [38,76].

### Histone Modifications in SLE

Unsurprisingly, histone modifications play a role in the pathogenesis of SLE. Global histone H3 and H4 hypoacetylation has been found in lupus CD4^+^ T cells [77], and aberrant histone modifications have been observed within the TNFSF7 promoter, leading to CD70 overexpression on T cells, which may contribute to the disease [78]. Treatment of healthy T cells with HDAC inhibitors resulted in decreased CD3ς chain expression, leading to abnormalities in T cells [79]. Tsokos *et al.* revealed that the transcription factor CREMα may participate in histone acetylation in active lupus T cells by silencing IL-2 expression through HDAC recruitment to Cre sites in the IL-2 promoter [80]. In addition to T cells, Dai *et al*. investigated an alteration of H3K4me3 in various key candidate genes in lupus PBMCs [81]. Global H4 acetylation was reportedly altered in lupus monocytes, and 63% of these H4 acetylated genes were potentially regulated by IFN regulatory factors [82].

On a more gene specific level, histone modification also regulates lupus-related cytokine secretion, such as enhanced H3 acetylation at the IL-17 locus and elevated IL-10 production by chromatin remodeling regulated by Stat3 [83,84]. Furthermore, histone hyperacetylation has been found to be associated with an increased level of TNF-α and enhanced maturation of lupus monocytes [85]. However, it has not been elucidated whether histone modifications represent the cause or the consequence of lupus, despite the fact that the involvement of histone modifications in pathogenesis has been further suggested by mouse studies. Sirtuin-1 (Sirt-1), a histone deacetylase, was found to be overexpressed in lupus-prone mice, MRL/*lpr* mice [86], and the down-regulation of Sirt-1 resulted in short-term enhanced H3 and H4 acetylation, with reduced levels of anti-dsDNA, glomerular IgG deposition and kidney damage [19]. Treatment of MRL/*lpr* mice with HDAC inhibitors also had therapeutic effects, with improvement of proteinuria, reduced renal damage and down-regulation of lupus-associated cytokine levels [87]. Recent progress has been made from a study that identifies candidate causal variants for 21 autoimmune diseases from genetic and epigenetic mapping in different subtypes of CD4^+^ T cells, including regulatory T cells, Th17, Th1 and Th2 cells [88]. In this outstanding study, distinct H3K27 peaks have been observed in the super-enhancer in IL2RA locus preferentially in regulatory T cells and Th17 cells.

The participation of histone modifications in the pathogenesis of SLE has been well documented. Nonetheless, given that histone hyper- or hypo-acetylation occurs in both region- and tissue-specific manners, and in light of the controversies mentioned above, the exact role of histone modification in the pathogenesis of SLE and the underlying mechanisms still require further study.

## 4. MicroRNAs (miRNAs) and SLE

**Figure 4:**
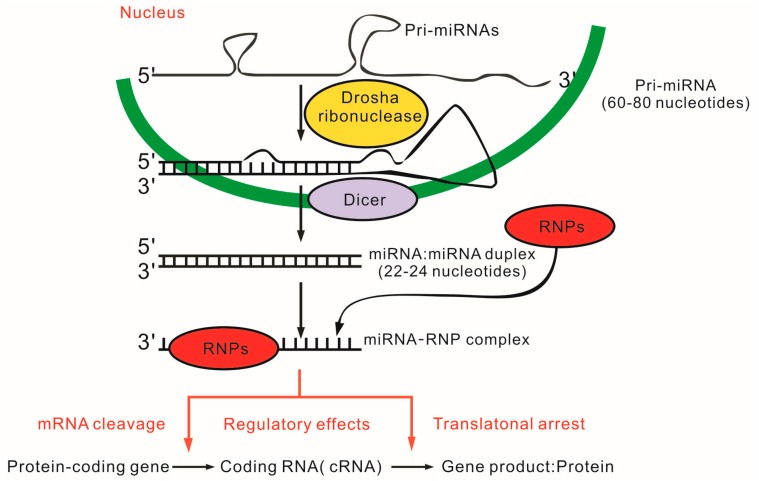
Gene regulation by miRNAs. miRNA genes are transcribed by RNA polymerase II in the nucleus to compose primary miRNAs (pri-miRNAs). The latter ones are recognized by nuclear enzymes, such as Drosha, forming ~70-nucleotide hairpin precursor miRNAs (pre-mRNAs). Mature miRNAs are cleaved from pre-mRNAs, by the enzyme Dicer, with a duplex form 18–23 nucleotides in length. One of these two strands with lower stability in the 5' end will be associated with the RNA induced silencing complex (RISC), the place that miRNAs bind to the mRNA targets. The predominant regulatory effect of miRNAs is to repress their target mRNAs.

miRNAs are small, non-coding RNAs (21–25 base pair) and function as posttranscriptional and posttranslational regulators of gene expression. MiRNAs perform their functions by binding to the 3'-untranslated region (UTR) of the messenger RNA (mRNA) of a target gene, causing mRNA cleavage, translational repression, or translational arrest [89,90] (Figure 4). Over 2000 miRNAs are registered in human miRNA databases, and up to 1000 of them control one-third of transcriptome, involving cell differentiation, cell cycle, apoptosis and immune responses [91,92,93]. Based on their functions, it is not surprising that they play important roles in the innate and adaptive immune systems, and thus may be relevant to the pathogenesis of SLE [94,95]. Research findings have shown the abnormal expression of miRNAs in different cell types and tissues in lupus, which may be associated with the progression of disease, making them the biomarkers for diagnosis and prognosis and even potential therapeutic targets or tools.

### 4.1. miRNAs in T Cells and SLE

It has been demonstrated that each miRNA may bind to different targets but regulate the same gene. Although numerous miRNAs are found to be abnormally expressed in T cells, certain miRNA target lupus-related gene expression, including IL-10, IL-17, and DNMT1. For example, miR-21, miR-126 and miR148a were found to be decreased in SLE T cells and to target DNMT1, though they were bound to different regions [96,97]. Interestingly, the repression of miR-21, miR-148a and miR-29b in SLE T cells was able to reverse pathogenic phenotypes to normal ones to some extent, suggesting their potential roles in SLE [97,98]. Moreover, miR-21 has been observed to suppress PDCD4 expression, thus enhancing cell proliferation and increasing CD40L and IL-10 expression in lupus T cells [99]. In addition, miR-142 [100] and miR-31 [101] have been reported to regulate T cell functions by inhibiting IL-4, IL-10, CD40L and ICOS expression and enhancing IL-2 production, respectively. Based on our previous finding for miR-146a and -241-3p/5p, we recently reported that mycophenolic acid, which has been applied in clinical use for lupus, ameliorates the autoreactivity of lupus T cells by regulating these two miRNAs, indicating the involvement of miR-146a and -241-3p/5p in SLE pathogenesis [102].

### 4.2. miRNAs in B Cells and SLE

Unlike the situation for T cells, relatively few studies have focused on miRNAs in regulating lupus B cells. Strikingly enhanced expression of miR-30a has been observed in lupus B cells, with a negative correlation with Lyn, which is a key negative regulator of B cell activation [103]. miR-155 and miR-181b have been reported to be negative regulators of activation-induced cytidine deaminase (AID), which regulates B cell antibody diversification [104,105]. In regulatory B cells, miR-15a has been observed to be positively correlated with the anti-dsDNA antibody level in an IFN-accelerated lupus mouse model [106]. Our recent finding reveals that miR-1246 in lupus B cells specifically targets EBF1 mRNA and therefore enhances B cell activation and functions [107].

### 4.3. miRNAs in Dendritic Cells and SLE

Dendritic cells (DCs) are divided into two distinct subsets: Plasmacytoid DCs (pDCs) and myeloid DCs (mDCs). pDCs have been recognized as an essential player in lupus with regard to type I IFN production upon TLR7/9 stimulation [65]. Type I IFNs have been described as the “IFN signature” of SLE, showing a high positive correlation between its inducible gene expression pattern and Systemic Lupus Erythematosus Disease Activity Index (SLEDAI) [108]. Recent studies have revealed a positive correlation of miR-146a with STAT1, which is involved in the type I IFN pathway, and polymorphism studies have confirmed its role in lupus [109,110,111]. Based on the findings that miR-155 [112] and other miRNAs [113] regulate pDC apoptosis and cytokine production, new evidence regarding the role of miRNAs in SLE may be brought to light in the near future.

### 4.4. Circulating miRNAs in SLE

Numerous circulating miRNAs have been identified to be correlated with lupus and have also been suggested to serve as biomarkers. Among them, miR-146a and miR-155 are the first-described miRNAs that are decreased in lupus serum and are regarded as biomarkers [114]. In subsequent studies, the serum levels of miR-200a, miR-200b, miR-200c, miR-429, miR-205, miR-192, miR-126, miR-16, miR-451, miR-223, miR-21, and miR-125a-3p [115,116] were found to be abnormally expressed in SLE and correlated with SLEDAI. More inspiring is that miR-126 has been reported to regulate DNA methylation in lupus T cells by targeting DNMT1 [96], supporting the idea that lupus T cells are switched on by DNA hypomethylation via miRNAs [117].

The aberrant expression of miRNAs in lupus is summarized in Table 2. However, the factors upstream and downstream of miRNA regulation are not well understood. Similar to DNA methylation, environmental and hormonal regulation are believed to be regulators of miRNAs in lupus. For example, air-pollution, especially containing metal-rich particles, increases the level of miR-21 [118], and miR-21 has been found to be enhanced in lupus and correlated with SLEDAI [99]. In addition, miR-146a is down-regulated in estrogen-treated splenocytes [119]. In general, miRNAs often do not perform their functions alone, and they act through their action on DNA methylation or even histone modifications to regulate immune responses in a synergistic fashion.

**Table 2:** Aberrant miRNA expressions in SLE.

Source miRNAs	Change	Reference
PBMCs: miR-21, miR-25, miR-146b, miR-155, miR-371-5p, miR-423-5p, miR-638, miR-663, miR-142-3p, miR-342, miR-299-3p, miR198	Increased	[99,120,121]
miR-125b, miR-342-3p, miR-146a, miR-196, miR-17-5p, miR-409-3p	Decreased	[120,122,123]
T cells: miR-224, miR-126, miR-21, miR-148a, miR-29b, miR-31	Increased	[96,99,124]
miR-145	Decreased	[124]
B cells: miR-1246, miR-15a	Increased	[106,107]
miR-30a, miR-155, miR181b	Decreased	[104,105]
DCs: miR-146a	Increased	[109,110,111]
Circulating: miR-142-3p, miR181a, miR-126, miR-16, miR-451, miR-223, miR-21	Increased	[115,116,125]
miR-146a, miR-155, miR-200a/b/c, miR-429, miR205, miR-192, miR-17, miR-20a	Decreased	[115,116,125]

## 5. Potential Epigenetic Therapies

Based on the findings in epigenetic mechanisms, the development and clinical use of HDAC inhibitors and anti-miRNA drugs for anti-inflammatory has launched a revolution in autoimmune diseases. HDAC inhibitors are reported to display anti-inflammatory and immunosuppressive effects [126,127], which may also have a benefit in SLE. HDAC inhibitors Trichostatin A (TSA) and subrroylanilide hydroxamic acid (SAHA) are the first two candidate therapies for SLE. TSA is reported to modify pro-inflammatory cytokine productions, such as IL-10, IFN-γ [36] and IFN-α [128]. Similarly, SAHA is found to inhibit TNF- α, IL-6 and NO [35]. Both of them showed therapeutic effects on MRL/*lpr* mice [28,35]. Moreover, ITF2357 is another candidate HDAC inhibitors that reduces the frequency of Th17 cells and increases regulatory T cells in NZB/W mice [129]. In addition, depletion of miRNAs, such as miR-155 [130] and miR-146a [114], results in reduced autoantibody levels, suggesting a therapeutic role in SLE.

## 6. Conclusions and Perspectives

Accumulating evidence suggests the involvement of epigenetic mechanisms in immune regulation and the pathogenesis of lupus. DNA hypomethylation, histone hypoacetylation and hyperacetylation, and decreased and/or enhanced expression of miRNAs have been identified to play a role in the pathogenesis of lupus. Moreover, recent studies have been focused on identifying the upstream and downstream factors that affect or impact epigenetic modifications. Efforts have been made, for example, to examine how environmental triggers promote epigenetic changes and contribute to lupus, explore the interaction of epigenetic modifications, and investigate the relationship between transcription factors and epigenetics. In a very recent report, 111 reference human epigenomes has been identified and analyzed, including 1821 histone modification data sets, 360 DNA accessibility data sets, 277 DNA methylation data sets, and 166 RNA-seq data sets [131], implying the real advent of the epigenomics era. Based on these findings, and future expected results, researchers will continue to broaden and deepen our understanding of pathogenesis of SLE and establish more individually optimized therapeutic strategies for lupus patients.
